# Neuroprotective and Neurological/Cognitive Enhancement Effects of Curcumin after Brain Ischemia Injury with Alzheimer’s Disease Phenotype

**DOI:** 10.3390/ijms19124002

**Published:** 2018-12-12

**Authors:** Ryszard Pluta, Marzena Ułamek-Kozioł, Stanisław J. Czuczwar

**Affiliations:** 1 Laboratory of Ischemic and Neurodegenerative Brain Research, Mossakowski Medical Research Centre, Polish Academy of Sciences, 02-106 Warsaw, Poland; mulamek@imdik.pan.pl; 2 First Department of Neurology, Institute of Psychiatry and Neurology, 02-957 Warsaw, Poland; 3 Department of Pathophysiology, Medical University of Lublin, 20-090 Lublin, Poland; czuczwarsj@yahoo.com

**Keywords:** brain ischemia, curcumin, Alzheimer’s disease, neurodegeneration, amyloid, tau protein, autophagy, mitophagy, apoptosis, genes

## Abstract

In recent years, ongoing interest in ischemic brain injury research has provided data showing that ischemic episodes are involved in the development of Alzheimer’s disease-like neuropathology. Brain ischemia is the second naturally occurring neuropathology, such as Alzheimer’s disease, which causes the death of neurons in the CA1 region of the hippocampus. In addition, brain ischemia was considered the most effective predictor of the development of full-blown dementia of Alzheimer’s disease phenotype with a debilitating effect on the patient. Recent knowledge on the activation of Alzheimer’s disease-related genes and proteins—e.g., amyloid protein precursor and tau protein—as well as brain ischemia and Alzheimer’s disease neuropathology indicate that similar processes contribute to neuronal death and disintegration of brain tissue in both disorders. Although brain ischemia is one of the main causes of death in the world, there is no effective therapy to improve the structural and functional outcomes of this disorder. In this review, we consider the promising role of the protective action of curcumin after ischemic brain injury. Studies of the pharmacological properties of curcumin after brain ischemia have shown that curcumin has several therapeutic properties that include anti-excitotoxic, anti-oxidant, anti-apoptotic, anti-hyperhomocysteinemia and anti-inflammatory effects, mitochondrial protection, as well as increasing neuronal lifespan and promoting neurogenesis. In addition, curcumin also exerts anti-amyloidogenic effects and affects the brain’s tau protein. These results suggest that curcumin may be able to serve as a potential preventive and therapeutic agent in neurodegenerative brain disorders.

## 1. Introduction

Brain ischemic injury in humans is the third cause of disability and the second cause of death, which may soon become the leading cause of full-blown dementia of Alzheimer’s disease type [1,2,3,4,5,6,7,8,9]. Unexpected cerebral ischemia refers to a global and local cerebral episode that causes sudden neurological deficits [10]. Recent epidemiological data indicate that approximately 17 million people suffer from ischemic brain damage every year [6,11]. The number of patients after ischemic cerebral episode has now reached 33 million [6,11]. According to epidemiological predictions, this figure will increase to 77 million in 2030 [11]. In addition, people with cerebral ischemia are at high risk of cognitive impairment. Physical impairment after ischemic stroke tends to improve to a greater or lesser extent. However, for some unknown reasons, the impairment of cognitive functions gradually progresses. Currently used treatment of made ischemic stroke involves the use of thrombolysis, but thrombolysis has a limited window of therapeutic time and the potential risk of hemorrhagic transformation [10]. Now, post-ischemic stroke has a huge impact on global public healthcare and clinical practice.

Patients with ischemic stroke frequently have cognitive deficits with varying degrees of differentiation [4,12]. Animals after experimental cerebral ischemia also show cognitive deficits [13,14,15,16]. Recently, the key role of episodic cerebral ischemia in the development of dementia has appeared at the forefront of clinical and experimental research [17,18,19,20,21,22,23,24,25,26,27,28]. Studies in recent years suggest that ischemic brain damage may promote neurodegeneration of the Alzheimer’s disease-type by damaging neuronal energy, generating reactive oxygen species [27,29], neuroinflammation [29,30,31,32], various parts of amyloid protein precursor accumulation [18,29,33], and tau protein dysfunction [34,35], which in turn damage neuronal cells, especially in the hippocampus and contribute to brain atrophy [33,36,37,38,39]. The vast majority of people who survived brain ischemia, experience progressive motor and cognitive deficits, which makes the development of new therapies to improve neurological outcomes even more urgent. Although many experimental studies have identified acute strategies to reduce the loss of nerve cell number during and after brain ischemia, only therapeutic hypothermia translates into use in a human clinic [40]. Therefore, due to the lack of translation of experimental neuroprotective agents for use in clinical conditions [41], we have focused our attention on improving functional outcomes after ischemia, instead of protecting neurons during ischemic damage. To this end, we should improve the function of persistent neurons after ischemia [14,16], and new therapies should be designed to reverse synaptic plasticity deficits to improve the functional outcome after brain ischemia, effectively extending the therapeutic window. This review presents current advances in the study of cerebral ischemia, focusing on ischemia-induced neurodegeneration of Alzheimer’s disease-type. It should be emphasized that despite the fact that brain ischemia is one of the leading causes of death and disability in the world, there is no effective treatment to improve the structural and functional consequences of this disorder. Therefore, in this review, we will also look at the promising role of the protective action of curcumin on the function and survival of persistent neurons after ischemic brain damage. Finally, we present the latest evidence that provides new information on the role and mechanisms of curcumin in inhibiting ischemia-reperfusion brain injury and potential therapeutic strategies in the treatment of ischemic brain damage of Alzheimer’s disease phenotype.

## 2. Similar Multifactorial Processes in the Post-Ischemic Brain and Alzheimer’s Disease

Post-ischemic brain damage is undoubtedly one of the most common multifactorial forms of neurodegeneration, including a series of abnormal cell/molecular processes taking place at different times during recirculation and progressively various areas of the brain. It seems that ischemic episodes favor the development of Alzheimer’s disease-like neurodegeneration through numerous mechanisms including neuronal loss, synaptic dysfunction, neuroinflammation, accumulation of various parts of the amyloid protein precursor, tau protein dysfunction and dysregulation of Alzheimer’s disease-related genes, white matter lesions, and general brain atrophy. The progress in understanding the key processes of brain ischemia-induced changes of the Alzheimer’s disease phenotype and genotype will help to develop the prevention and treatment strategies against neurodegeneration and dementia generated by ischemia.

### 2.1. Neurodegeneration after Brain Ischemia

In the hippocampus, necrotic and apoptotic neuronal cells were observed in the CA1 region 2–7 days after brain ischemia [29,33,42]. After two days of recirculation, the loss of neurons was superimposed with damaged nerve cells. In later times of observations up to six months, the number of neurons with pathological changes has been reduced and replaced by the loss of nerve cells. The above changes were mainly located in the hippocampus and third, fifth, and sixth layer of the cortex. The borderline zones of the brain cortex were also the site of serious neuropathological changes. Over six months after ischemic brain damage, apart from the local loss of neurons, various types of pathological neuronal changes have been observed. The first of these was the form of chronic neuronal degeneration, which was observed in the early periods following brain ischemia. Other changes were typical of early ischemic lesions but were observed in those areas of the brain that were not involved in early changes, such as the hippocampus region CA2, CA3, and CA4 [29,33,42]. The death of neurons in the CA1 region of the hippocampus, along with the decrease in the acetylcholine level in the cerebral cortex and striatum, is evident after cerebral ischemia, suggesting that the loss of neurons may result from the insufficiency of neuronal excitable transmission [43,44].

The synaptic integrity of the brain is essential for physiological activities, including memory and learning. Decreased levels of both synaptophysin and postsynaptic density protein 95 were found in the rat hippocampus after local cerebral ischemia [45,46]. Similarly, these rats presented ultrastructural synaptic changes in the CA1 region of the hippocampus. Other studies have shown that ischemic brain damage leads to an increase in both synaptic autophagy and asymmetric synapses that may be associated with the death of neurons in the CA1 region of the hippocampus after transient cerebral ischemia [47,48,49]. There is experimental evidence of an isolated and persistent abnormal synaptic function resulting from transient cerebral ischemia [50]. In the CA1 region of the hippocampus, depression of excitatory synaptic transmission after brain ischemia has been demonstrated [43]. Ischemia-induced increase in intracellular Ca^2+^ regulates the activity of calpain in neurons, and calpain target proteins are present in GABAergic and glutaminergic synapses. In brain ischemia, calpain cleaves pre- and postsynaptic proteins. The distribution of protein cleavage via calpain contributes to neuronal death in brain ischemia [51].

The damage of the white matter and the activation of glial cells are observed both in animals and in people after brain ischemia [29,32,36,37,42,52,53,54]. In models of rat brain ischemia, it seems that ischemia causes more serious changes in the subcortical white matter and corpus callosum [29,36,37,55]. These findings are consistent with glial activation in the corpus callosum following ischemia-reperfusion brain injury [56]. Brain ischemia favors the increase of blood–brain barrier permeability, which facilitates the penetration of inflammatory cells into the brain parenchyma and releases a large number of serine proteases, as well as β-amyloid peptides and tau protein from the blood into the brain tissue, which in turn leads to white matter lesions [18,32,57,58,59,60,61,62,63,64,65,66].

Evidence suggests that transient ischemic brain damage in rats causes extensive loss of neurons, in structures belonging to or not to selectively sensitive areas of the brain [29,42]. Ischemic changes in the brain represent a gradually progressive process that stretches for a long time during reperfusion after an ischemic episode [29]. They are characterized not only by early changes in the brain, but also by an active pathological process in the late stage following ischemic injury. Within one to two years after cerebral ischemia, the neuropathological process leads to generalized brain atrophy [29,42,67]. Brain gross examination, carried out from 9 to 24 months following ischemia-reperfusion episode, showed symptoms of brain hydrocephalus [42,67]. Dilatation of the subarachnoid space around the cerebral hemispheres was also observed [42]. Complete disappearance of the hippocampus with massive pyramidal neuron loss in its CA1 area and atrophy of the striatum was noted [42]. The brain cortex was narrow, which suggested an increased density of neurons. An additional feature of late atrophy of the brain was manifested as scattered changes of the white matter, taking the form of rarefaction and cavitations, manifesting as advanced spongiosis. This phenomenon can be explained by the huge loss of neurons along with the ischemic acute and chronic increased permeability of the blood–brain barrier [48], for example for neurotoxic amyloid and tau protein [57,58,59,61,63,64,65,66].

### 2.2. Amyloid Generation after Brain Ischemia

Amyloid deposits occur in both the brain parenchyma and the vascular walls of the brain after ischemic episodes in humans and animals [19,29,68,69,70,71,72,73]. Animals that survived up to seven days after ischemic brain injury showed intense brain immunoreactivity for the N-terminal of the amyloid protein precursor, the β-amyloid peptide, and for the C-terminal of the amyloid protein precursor. In animals that survived up to one year after ischemic brain injury, increased staining was reported only for the C-terminal of the amyloid protein precursor and the β-amyloid peptide [29,30,31,42]. Staining was observed intra- and extracellular [19,29]. Extracellular different parts of the amyloid protein precursor deposits ranged from scattered small dots to irregularly dispersed diffuse plaques [19,29,74,75,76,77,78]. In the hippocampus, entorhinal cortex, corpus callosum, around lateral ventricles and in the thalamus multifocal scattered amyloid plaques were observed. The time-dependent accumulation of the β-amyloid peptide in the hippocampus, especially in fields with the open blood–brain barrier, occurred after experimental brain ischemia [18,36,37,45,62,63,79,80]. It should be emphasized that β-amyloid peptide deposits observed after experimental cerebral ischemia did not stain with thioflavin S [19,81], while in humans some amyloid deposits were stained with thioflavin S [69]. Accumulation of amyloid in the vessel walls caused by ischemic brain damage with vasospasm may additionally cause ischemic changes and develop a self-propelling vicious cycle of ischemic episodes, ultimately leading to irreversible damage to the brain parenchyma [29,82].

### 2.3. Dysfunction of Tau Protein after Brain Ischemia

The microtubule-associated tau protein is hypophosphorylated in the ischemic brains of patients and in experimental cerebral ischemia and ultimately generates intraneuronal neurofibrillary tangles and/or neurofibrillary tangle-like tauopathy that are a key in the ongoing neuropathology of Alzheimer’s disease [35,83]. Cyclin-dependent kinase 5 is involved in neurofibrillary tangle-like tauopathy, which is caused by ischemic hyperphosphorylation of tau protein [34]. Also, phosphorylation of tau protein in many places specific for Alzheimer’s disease caused by experimental cerebral ischemia was observed [46]. Increased phosphorylation of tau protein with cyclin-dependent kinase 5 parallel activation, glycogen synthase kinase-3b and calcium/calmodulin dependent protein kinase II, as well as inhibition of protein phosphatase 2A have been reported after focal rat brain ischemia [84]. It can therefore be suggested that increased phosphorylation of tau protein and overproduction of β-amyloid peptide appear to be very sensitive to brain ischemic injury.

### 2.4. Dysregulation of Genes Associated with Alzheimer’s Disease after Brain Ischemia

In the CA1 area of hippocampus, the expression of the amyloid protein precursor gene was lowered below the control value within two days after ischemic brain injury [85,86]. Seven and 30 days after cerebral ischemia, the expression of the amyloid protein precursor gene increased above the control value [85,86]. In the temporal cortex, the expression of the amyloid protein precursor gene has been lowered below the control value two days after ischemic brain injury [87]. However, on days 7 and 30 after brain ischemia, the expression of the amyloid protein precursor gene was increased above the control value [87]. Expression of the β-secretase gene increased above the control value after cerebral ischemia in the rat hippocampal CA1 area two to seven days after the injury [85,86]. However, 30 days after cerebral ischemia, expression of the β-secretase gene decreased below the control value [85,86]. Expression of the β-secretase gene was regulated upward in the temporal cortex two days after brain ischemia [87]. Seven and 30 days after temporal cortex ischemia, the expression of the β-secretase gene was significantly reduced [87]. In the hippocampal CA1 region, the expression of the presenilin 1 and 2 gene was above the control value two and seven days after brain ischemia [85,86]. However, 30 days after an ischemic injury, the gene expression of presenilin 1 and 2 decreased below the control value [85,86]. In the temporal cortex, presenilin 1 gene expression decreased below the control value, but presenilin 2 increased above control 2 days after ischemic brain injury [88]. Seven days after cerebral ischemia, gene expression of presenilin 1 was reduced, and presenilin 2 was significantly elevated [88]. Thirty days after the termination of cerebral ischemia, the expression of presenilin 1 gene increased above the control value and presenilin 2 decreased below the control [88].

There is only one study in the literature indicating the relationship between the ischemic CA1 area of the hippocampus and the expression of the tau protein gene following transient brain ischemia in rats with 2, 7, and 30 days survival [35,89]. In the CA1 area of the hippocampus, the expression of the tau protein gene increased approximately 3-fold with respect to control values on the second day following brain ischemia [89]. On the 7th and 30th day after brain ischemia, the expression of the tau protein gene oscillated in the range of control values [89]. Statistically significant changes in the expression of the tau protein gene after brain ischemia were between 2 and 7 and 2 and 30 days of survival [89].

It was found that the autophagy gene in the hippocampal CA1 region was not significantly modified 2, 7, and 30 days after ischemic brain injury [90]. However, the mitophagy gene was significantly elevated on day 2 and fell below baseline on days 7 and 30 [90]. Expression of the caspase 3 gene in the CA1 region of the hippocampus two days after brain ischemia increased by more than 300% compared to baseline. However, seven days after ischemic injury, its expression was close to its basic value. Thirty days after ischemic injury, the gene expression was lowered below baseline in the above area [90]. In the temporal cortex, gene expression of autophagy increased within 2–30 days after transient brain ischemia in rats [91]. However, the gene of mitophagy fell below the normal value within two days after brain ischemia. Seven and 30 days after cerebral ischemia, the expression of the mitophagy gene increased above control values. Expression of the apoptotic caspase 3 gene was reduced below normal values two days after brain ischemia. Seven and 30 days after ischemia, the expression of caspase 3 gene increased above control values [91].

### 2.5. Behavioral Changes after Brain Ischemia with Alzheimer’s Disease Phenotype

After brain ischemia in animals, behavioral abnormalities were also observed in addition to neurodegenerative changes [13,14,15,16,92,93]. It should be emphasized that neurodegenerative changes after ischemia do not cause noticeable long-term neurological deficits in animals [92]. After the ischemic episode, a spontaneous return of the sensory-motor function in animals was observed [14,94,95]. After ischemic brain injury, excessive locomotor activity was noted [96,97], as in people with Alzheimer’s disease. Longer brain ischemia causes longer locomotor hyperactivity [32,63,92,98]. An impairment of habituation was observed after ischemic brain injury, which results in longer exploration time [99,100]. Brain ischemia causes a reference and working memory deficits [14,101,102]. In addition, ischemic brain damage in animals gradually leads to deficits in spatial memory during post-ischemic survival [14,103,104]. Progression of cognitive impairment has been demonstrated at different times during recirculation [14,104,105]. In addition, evidence of recurrent ischemic brain injury in animals showed persistent locomotor hyperactivity, severe cognitive deficits, and reduced level of anxiety [106]. Vigilance and sensory-motor efficiency are damaged for one or two days, while deficits in learning and memory seem to be irreversible and indefinitely persistent [14,98]. The aforementioned behavioral changes were associated with the loss of neurons in the CA1 region of the hippocampus, cerebral cortex, caudate nucleus [29,63,106], amygdala and perirhinal cortex [13], and with significant brain atrophy [29,30,31,42,107].

## 3. Effect of Curcumin on Neurodegenerative Changes and Neurological/Cognitive Function after Brain Ischemia

### 3.1. Neuroprotective Effects

The administration of curcumin before reperfusion in the model of middle cerebral artery occlusion in rats reduced the size of cerebral infarction and cerebral edema (Table 1) [108]. Before reperfusion after middle cerebral artery occlusion, treatment with curcumin reduced neutrophil rolling and adhesion to the endothelium of the cerebrovascular system by 76% and 67%, respectively. Because neutrophils are the main source of oxidant damage during reperfusion, curcumin blocks the major contributing factor to reperfusion injury, preventing neutrophil attack and accumulation in ischemic sites after experimental brain ischemia [108]. Curcumin reduced reperfusion injury in ischemic stroke by preventing neutrophil adhesion to cerebrovascular microcirculation [108]. In the rat model of embolic stroke, the efficacy of curcumin after ischemia was demonstrated, in which curcumin reduced infarct volume, improved sensory motor function, and significantly reduced the stress associated with nitrosis [109]. Curcumin can protect against local ischemic brain damage with reperfusion and also stimulate neurogenesis by activating the Notch signaling pathway [110]. There was also a clear decrease in the apoptotic index after three days of reperfusion in groups receiving curcumin. Significantly more TUNEL-positive neuronal cells were found in the ischemic group compared to curcumin-treated ischemic groups [111]. In addition, Kalani et al., [112] have shown that embryonic stem cell exosomes loaded with curcumin reduced astrogliosis and improved neuronal survival after brain ischemia in mice. Embryonic stem cell exosomes loaded with curcumin restored the neurovascular unit after ischemic brain injury [112,113]. These results suggest that combining exosomal potentials from embryonic stem cells with curcumin may help restore the neurovascular unit after ischemic brain injury in mice. All acute therapies with curcumin reduced the activity of matrix metalloproteinase-9 and hemorrhagic transformation of ischemic stroke in diabetic rats [113]. In addition, curcumin has reduced cerebral edema in these animals. Administration of curcumin for two months significantly reduced the ischemia-induced death of neurons in the CA1 region of the hippocampus, as well as impaired glial activation [114]. Administration of curcumin in the above condition also reduced lipid peroxidation, mitochondrial dysfunction, and apoptotic indices. After intranasal administration of curcumin, locomotor activity, and reduction in grip strength were improved after middle cerebral artery occlusion [115].

### 3.2. Neurological/Cognitive Effects

Treatment with curcumin also improved neurological outcomes after focal cerebral ischemia [108]. Administration of curcumin after ischemia improved sensory-motor activity [109]. Rats with focal cerebral ischemia treated with curcumin showed significantly smaller neurobehavioral deficits than animals treated with vehicle after 3, 7, and 12 days of reperfusion [110]. After one and three days, a significant reduction in neurological score was noted in the curcumin-treated groups compared to the control ischemic group [111,112]. Improvement in neurological function was observed, as evidenced by gait results, modified Bederson’s scores and grip strength, but the size of the infarct was similar to untreated animals with ischemia and diabetes [112,113]. Biochemical changes resulting from the administration of curcumin also correlated very well with the ability to relieve changes in locomotor activity after ischemia-reperfusion brain injury [114,115].

## 4. Possible Molecular Mechanisms Underlying the Protective Action of Curcumin after Ischemia-Reperfusion Brain Injury

Apoptosis is one of the main routes that lead to the process of neuronal cell death after brain ischemia [122]. Curcumin contributes to the protection of neurons, probably through anti-apoptotic mechanisms [123]. Curcumin increased the level of anti-apoptotic Bcl-2 protein in the mitochondria and reduced the subsequent translocation of cytochrome c to the cytosol, thereby weakening the activation of caspase [123]. It is suggested that the mitochondrial pathway is an important target for curcumin. Curcumin administration has been shown to completely inhibit ischemia-induced cytochrome c release [114]. Another mechanism by which curcumin prevents damage to the ischemic brain is to increase the expression of the silent information regulator 1, a key neuroprotective molecule that participates in protection against ischemia-reperfusion brain damage. In this respect, the activation of the silent information regulator 1 leads to deacetylation of p53 and attenuation of apoptosis in the brain after ischemia [124]. It has been documented that the number of mitochondria and their mass, mitochondrial biogenesis and mitochondrial uncoupling protein 2 are significantly reduced in rats with ischemic brain injury, and these changes are reversed by pre-treatment with curcumin. It was also shown that mitochondrial biogenesis was increased in the focal model of brain ischemia in rats after administration of curcumin [125]. The data suggest that curcumin can alleviate ischemia-reperfusion injury of the brain by preventing ONOO^−^-induced damage to the blood–brain barrier, and this indicates that curcumin alleviates vasogenic edema of the brain by protecting the integrity of the blood–brain barrier [118].

It is suggested that in response to ischemia-reperfusion injury of the brain, numerous factors predisposing to stress from the endoplasmic reticulum are activated in neurons, including depletion of the endoplasmic reticulum of Ca^2+^, proteins aggregation, reduced proteins degradation, and accumulation of lipid peroxidation products in the endoplasmic reticulum and structures of Golgi apparatus [126]. Endoplasmic reticulum stress can induce proapoptotic processes and lead to apoptosis [127,128]. The growth arrest- and DNA damage-inducible gene 153 and caspase 12 are among the main factors of apoptosis mediated by stress of the endoplasmic reticulum [129]. Growth arrest- and DNA damage-inducible gene 153 is a signaling molecule that is involved in the development of apoptosis via pathways, such as the effect on intracellular Ca^2+^ metabolism and a reduction in Bcl-2 [129,130,131]. Caspase-12 is released from the endoplasmic reticulum during stress of the endoplasmic reticulum, and then activates the caspase cascade and apoptosis. It has been observed that curcumin can reduce the stress of the endoplasmic reticulum by reducing the expression of growth arrest- and DNA damage-inducible gene 153 and caspase-12, thus exhibiting a protective effect against brain ischemia-reperfusion injury in animals [129].

In the early stages after ischemic damage to the brain, neuroinflammation accelerates the damage process and determines the degree of brain damage [32,132]. Oxidative stress and overproduction of reactive oxygen species is a permanent element and an important mechanism of brain damage after ischemia-reperfusion [133]. Available studies have shown that the administration of curcumin prevents ischemia-reperfusion injury due to its antioxidant activity [115,134,135,136,137]. Possible mechanisms for the protective effect of curcumin on oxidative stress include reduction of lipid peroxidation, increased protein synthesis, free radical scavenging, increased glutathione content, and maintenance of cell membrane integrity [115,137,138]. It was shown that the increase in peroxiredoxin 6 level by curcumin weakened ischemic oxidative damage by induction of factor specific protein 1 in post-ischemic rats [137]. Ischemic in vitro studies have shown that curcumin increases thioredoxin levels and protects neurons from death due to deprivation of oxygen and glucose [139]. In addition, curcumin protects the brain against ischemia-reperfusion injury by inhibiting neuroinflammatory cytokines, such as IL-6 and TNF-α [124].

It was found that the antioxidant and anti-inflammatory action of curcumin contributed to the reversal of cognitive deficits related to the neurotoxicity of the β-amyloid peptide [140]. In experimental models both in vivo and in vitro, it has been shown that curcumin reduces the level of soluble β-amyloid peptide and the density of β-amyloid peptide plaques in brain tissue [141,142]. It has been revealed that curcumin prevents aggregation of the β-amyloid peptide in vitro and promotes the clearance of aggregates of the β-amyloid peptide. In addition, it has been presented that curcumin inhibits the maturation of the amyloid protein precursor and inhibits the generation of β-amyloid peptide in vitro [142]. Curcumin—by reducing the level of soluble tau protein, increasing the heat shock protein associated with the removal of tau protein, even after the formation of tangles—indicates that synaptic and behavioral dysfunctions caused by the tau protein are reversible [143]. It has been shown that curcumin inhibits oligomerization of the β-amyloid peptide and phosphorylation of tau protein in brain parenchyma and reverses cognitive deficits in the Alzheimer’s disease model [144]. Curcumin has been documented to stimulate neuronal stem cell proliferation and neuronal differentiation and reverses β-amyloid peptide-induced inhibition of hippocampal neurogenesis and memory deficits in the Alzheimer’s disease model [144,145]. Curcumin reduced the level of amyloid deposits and inhibited tau protein aggregation in the transgenic model of Alzheimer’s disease and reduced oxidative damage, neuroinflammatory, and neurological response, as well as cognitive deficit after infusion of amyloid into the brain [146]. Curcumin reduces the level and activity of beta-secretase, aggregation of beta-amyloid peptide and accumulation, and increases amyloid clearance [147]. Curcumin also induces amyloid uptake by macrophages and stimulates metal chelation [147]. A summary of protective action of curcumin after brain ischemia injury is presented in Table 1.

## 5. Conclusions

In the present review, we discussed the neuroprotective effects of curcumin after ischemia-reperfusion brain injury. Accumulating evidence has clearly shown the role of the neuroprotective and neurological/cognitive enhancement effects of curcumin after brain ischemia-reperfusion injury with the phenotype of Alzheimer’s disease (Table 1). Based on the data presented, it appears that curcumin has its own effective therapeutic potential through anti-amyloid, anti-tau protein hyperphosphorylation, anti-hyperhomocysteinemia, anti-oxidant, anti-inflammatory, and anti-apoptotic effects (Table 1) [116,117,119,143], which clearly indicates that curcumin can be used as a neuroprotective substance not only in ischemic neurodegeneration [33,148] but also in a neurodegenerative disease similar to Alzheimer’s disease as a response to brain ischemia associated with hyperhomocysteinemia [149,150]. The available data show that curcumin induces neuroprotection and neurogenesis and may be a new therapeutic agent for both regenerative medicine and for the treatment of neurodegenerative disorders such as neurodegeneration after brain ischemia with the phenotype of Alzheimer’s disease. Therefore, curcumin may be a promising supplementary agent against brain ischemia-reperfusion injury in the future. Indeed, there is a rational scientific basis for the use of curcumin for the prophylaxis and treatment of ischemic neurodegeneration. Nevertheless, despite initial hard data, prospective studies are needed to further clarify how curcumin could exert protective action against ischemic brain damage and how it can be used therapeutically. In particular, evidence from clinical randomized controlled trials would be helpful.

## Figures and Tables

**Table 1:** Summary of the protective action of curcumin on post-ischemic brain damage.

Kind of Ischemia	Animal	Treatment Time	Protective Action	References
Focal brain ischemia	Rat	Pre-treatment	Reduction of reperfusion injury by preventing neutrophil adhesion to the brain microcirculation	[108]
Incomplete brain ischemia	Rat	Pre-treatment	Inhibition of mitochondrial ROS generation, lipid peroxidation and neuro-protection by inhibiting apoptosis	[116]
Forebrain ischemia	Rat	Pre-treatment and post-ischemia	Reduction of apoptosis	[111]
Focal brain ischemia	Rat	Post-ischemia	Reduction in volume of infarct and brain edema	[117]
Focal embolic ischemia	Rat	Post-ischemia	Reduction in volume of infarct and improvement of sensory motor activity	[109]
Focal brain ischemia	Mouse	Post-ischemia	Reduced volume of infarct, brain edema, and blood–brain barrier permeability	[118,119]
Focal brain ischemia	Rat	Post-ischemia	Stimulation of neurogenesis and smaller neurobehavioral deficits	[110]
Forebrain ischemia	Gerbil	Post-ischemia	Reduction of neuronal death, glial activation, apoptotic indices and mitigation of changes in locomotor activity	[114]
Focal brain ischemia	Rat	Post-ischemia	Reduction of hemorrhagic transformation, brain edema and improvement of neurological function	[113]
Focal brain ischemia	Mouse	Post-ischemia curcumin-loaded mouse embryonic stem cell exosomes	Reduction of neurological score, infarct volume, edema, inflammation and astrogliosis and restoration of neurovascular system	[112]
Focal brain ischemia	Rat	Post-ischemia	Reduced neurological score, infarct area, apoptosis, caspase-3 mRNA expression and autophagy activity	[120,121]
